# Primary Clear Cell Microcystic Adenoma of the Sinonasal Cavity: Pathological or Fortuitous Association?

**DOI:** 10.1155/2017/9236780

**Published:** 2017-02-05

**Authors:** Rosalin Cooper, Hannah Markham, Jeffery Theaker, Adrian Bateman, David Bunyan, Matthew Sommerlad, Gillian Crawford, Diana Eccles

**Affiliations:** ^1^Department of Cellular Pathology, University Hospital Southampton NHS Foundation Trust, Southampton, UK; ^2^Wessex Regional Genetics Laboratory, Salisbury NHS Foundation Trust, Salisbury, UK; ^3^University of Southampton, Southampton, UK; ^4^Wessex Clinical Genetics Service, University Hospitals Southampton NHS Foundation Trust, Southampton, UK

## Abstract

Primary clear cell microcystic adenoma of the sinonasal cavity is rare. It has previously been described only as a VHL-associated tumour. Von Hippel-Lindau (VHL) syndrome is an inherited cancer syndrome characterised by an elevated risk of neoplasia including clear cell renal cell carcinoma (ccRCC), haemangioblastoma, and phaeochromocytoma. We describe the second reported case of a primary clear cell microcystic adenoma of the sinonasal cavity. The 39-year-old patient with VHL syndrome had previously undergone resection and ablation of ccRCC. He presented with epistaxis. Imaging demonstrated a mass in the ethmoid sinus. Initial clinical suspicion was of metastatic ccRCC. However, tumour morphology and immunoprofile were distinct from the previous ccRCC and supported a diagnosis of primary microcystic adenoma. Analysis of DNA extracted from sinonasal tumour tissue did not show loss of the wild-type allele at the* VHL* locus. Although this did not support tumour association with VHL disease, it was not possible to look for a loss-of-function mutation. The association of primary microcystic adenoma of the sinonasal cavity with VHL disease remains speculative. These lesions are benign but are likely to require regular surveillance. Such tumours may require repeated surgical excision.

## 1. Introduction

Von Hippel-Lindau (VHL) disease is a cancer syndrome characterised by an increased risk of multiple tumour types occurring across different organ systems. These include haemangioblastomas within the central nervous system and characteristic visceral lesions including clear cell renal cell carcinoma (ccRCC) and phaeochromocytoma [1]. Microcystic pancreatic lesions can also occur in the context of VHL disease [2]. Affected individuals carry germline mutations in the* VHL* tumour-suppressor gene with loss of the wild-type allele (normal gene copy) in a VHL disease-associated organ system leading to tumour formation [1, 3].

Sinonasal tumours are rare [4, 5]. Common benign lesions include inverted papillomas and osteomas [4], whilst common malignant lesions include squamous cell carcinoma and adenocarcinoma [5]. There has been only one reported case of a primary clear cell microcystic adenoma of the sinonasal cavity in the literature [6]. In this previously reported case, molecular analysis of tumour DNA confirmed tumour VHL disease association. Here we describe the second reported case of primary clear cell microcystic adenoma of the sinonasal cavity.

## 2. Case Report

### 2.1. Clinical History

A 39-year-old man with molecularly confirmed VHL disease presented with epistaxis in 2012. Molecular analysis had confirmed a germline* VHL* mutation in 2004. He had previously undergone excision of cerebellar haemangioblastoma in 2003. He had also undergone bilateral nephron sparing surgery for ccRCC (stage pT1a, Fuhrman grade up to 3) in 2005 and renal radiofrequency ablation in both 2008 and 2012. CT imaging demonstrated an ethmoid mass measuring 35 × 34 × 44 mm. The patient underwent endoscopic transsphenoidal resection of the lesion. Staging investigations were negative for metastatic disease.

Two years later, in 2014, the patient developed symptoms of nasal obstruction. Sinonasal tumour recurrence was suspected. Imaging demonstrated limited progression in the nasal cavity. Excision biopsy was performed. Histology appeared consistent with the initial presenting sinonasal tumour. Excision appeared complete, with no evidence of residual tumour. Subsequently, symptoms recurred. Therapeutic excision was performed in 2015 and again in 2016. To date, the patient's VHL disease remains stable with no evidence of new lesions or metastatic disease.

The patient remained under the management of specialist multidisciplinary (MDT) team which coordinated regular imaging surveillance and clinical review. The MDT recommended regular magnetic resonance imaging (MRI) surveillance with localised resection as applicable. More extensive surgery or adjuvant therapy was not felt to be indicated.

### 2.2. Histology

Slides of the sinonasal tumour resected in 2012 and the patient's ccRCC resected in 2005 were collated to allow comparison. Immunohistochemical and special stains for CD10, RCC, CK7, CK20, epithelial marker AE1/3, vimentin, EMA, Ki67, ssms1, sma, alpha-inhibin, p63, thyroglobulin, NSE, s100, GFAP, and PAS were performed according to standard automated protocols (Dako, UK).

**Figure 1:**
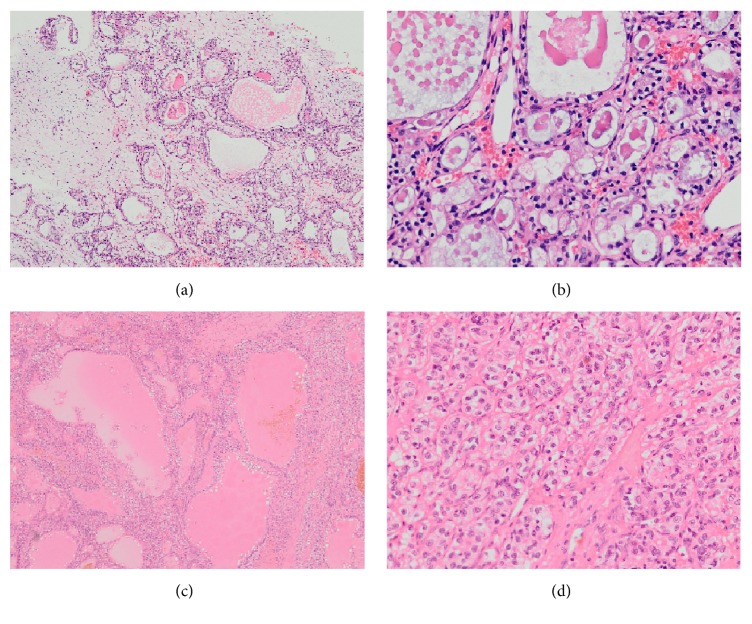
Haematoxylin and eosin (HE) stains of the sinonasal tumour (a) ×4 and (b) ×20 and renal tumour (c) ×4 and (d) ×20.

The sinonasal tumour demonstrated tubulocystic morphology with a brush border, glycogen-rich cells, and low grade nuclei without conspicuous mitoses (Figures 1(a) and 1(b)). The renal tumour consisted of clear tumour cells with tubulopapillary morphology with hobnailing at the luminal surface with Fuhrman grade up to 3 (Figures 1(c) and 1(d)).

**Figure 2:**
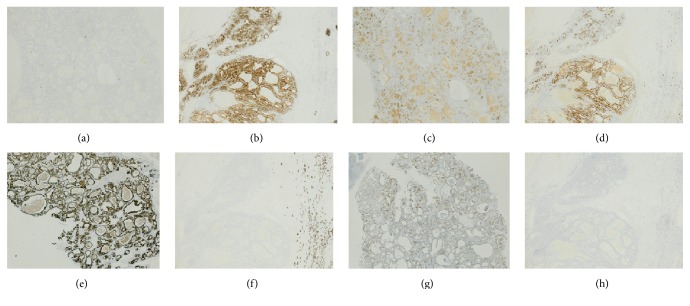
Immunohistochemistry (IHC) of renal and sinonasal tumours, ×2. (a) Sinonasal tumour cells were negative for CD10. (b) Renal tumour cells were strongly positive for CD10. (c) Sinonasal tumour cells were negative for RCC; however, weak staining of luminal secretions was seen. (d) Renal tumour cells demonstrated strong positivity for RCC marker. (e) Sinonasal tumour cells demonstrated positivity for CK7. (f) The renal tumour was negative for CK7; however, in this image a proximal renal tubule was seen to exhibit CK7 positivity. (g) The sinonasal tumour exhibited patchy CK20 positivity. (h) The renal tumour was negative for CK20.

**Figure 3:**
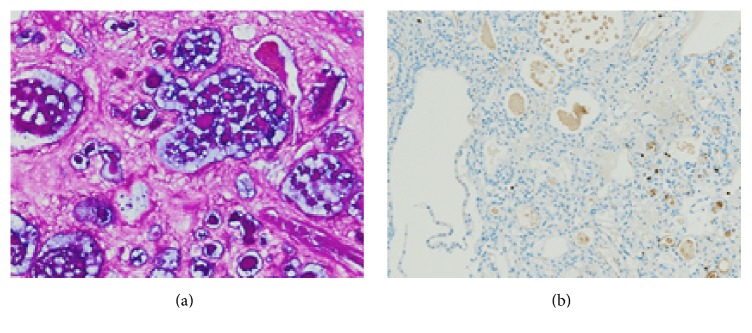
(a) Sinonasal tumour with luminal PAS-positive secretions, ×20. (b) Ki67 staining demonstrates a low mitotic index, ×10.

The sinonasal tumour was positive for CK7, epithelial marker AE1/3, vimentin, NSE, and EMA, with patchy CK20 staining, but negative for RCC and CD10. Secretions were PAS-positive and Ki67 demonstrated a low proliferation index. Immunohistochemistry revealed renal tumour positivity for RCC, CD10, and EMA and negativity for CK7 and CK20 (Figures 2 and 3 and Table 1).

### 2.3. *VHL* Sequencing Analysis

The patient was known to have a constitutional deletion of exon 1 of* VHL* confirmed by germline DNA analysis in 2004. DNA extracted from the sinonasal tumour resected in 2012 underwent molecular analysis in 2015 for loss of heterozygosity (LOH) at the* VHL* locus.

Point mutation analysis of the three coding exons of* VHL* (Genbank accession number NM_000551.3) was carried out by direct sequencing analysis of sinonasal FFPE-extracted tumour DNA. PCR products were generated using a PCR reaction with a 25 *μ*l volume and a 60°C annealing temperature using exon primers; 1F-tccgacccgcggatccc, 1R-tcagaccgtgctatcgtcc, 2F-gacgaggtttcaccacgtta, 2R-tcaagtggtctatcctgtact, 3F-tcgttccttgtactgagacc, and 3R-gtaccatcaaaagctgagatg. For the exon 1 fragment 5 *μ*l of Q solution was added to the PCR reaction (Qiagen, Manchester, UK). Products were sequenced using the Big-Dye® Terminator v1.1 Cycle Sequencing Kit standard protocol (Applied Biosystems, USA) and separated on an ABI 3130*xl* Genetic Analyzer (Applied Biosystems, USA). Data was analysed using Mutation Surveyor (version 3.1) software (SoftGenetics, USA). Dosage analysis was attempted using the Multiplex Ligation-dependent Probe Amplification (MLPA) method [7], but this failed due to sample quality.

Analysis of tumour DNA demonstrated no causative mutations; however, the tumour was heterozygous for an intron 1 polymorphism (c.341-50G>A), reducing the likelihood of LOH.

## 3. Discussion

Primary tumours of the nasal cavity are rare [4, 5]. Metastases from other primary tumour sites, whilst unusual, may also occur in this region, including those from a primary ccRCC [8–10]. To date, there has been one reported case of a primary clear cell microcystic adenoma occurring within the sinonasal cavity. This occurred in the context of VHL disease [6].

In the case we present here the initial clinical suspicion was of metastatic ccRCC. Histologically, the clear tumour cells, cystic morphology, and positive staining for AE1/3 [11] and vimentin [12] were supportive of this diagnosis. However, CD10 and RCC negativity, both of which were expressed by the previously resected renal tumour, was not consistent with this interpretation [13]. The tubulocystic morphology, brush border, and presence of PAS positive secretions were also distinct from ccRCC. Furthermore, the tubulocystic morphology, low Ki67 index, and absence of conspicuous mitoses were dissimilar from a primary sinonasal clear cell carcinoma. Sinonasal clear cell carcinomas are rare. These tumours tend to form sheets without glandular structures [14]. They can be locally invasive [15]. Such tumours may also metastasize [15].

In the previously reported case of a primary clear cell microcystic adenoma of the nasal sinus, Xu et al. describe microcystic tumour morphology, clear glycogen-rich cells without nuclear atypia, EMA and cytokeratin positivity, and negativity for CD10 [6]. These features are consistent with those of the case presented here. Similarity was also noted between features of the sinonasal tumour and those of pancreatic microcystic adenoma. Typically these pancreatic tumours, which can be both VHL-associated and sporadic, are characterised by cystic morphology, glycogen-rich cytoplasm, a low mitotic index, and AE1/3 positivity [2]. These are benign lesions [2]. Conventionally, small lesions in asymptomatic patients managed conservatively with regular imaging surveillance. Surgical resection is considered for larger or symptomatic tumours [16]. It is possible that the primary microcystic adenoma of the sinonasal tract is a similar tumour entity.

Xu et al. demonstrated sinonasal microcystic adenoma* VHL* LOH [6]. This confirmed tumour association with VHL disease [6]. In the case we describe here the sinonasal tumour was heterozygous at the* VHL* locus. The presence of an intron 1 polymorphism makes LOH unlikely. Therefore, molecular analysis did not support tumour association with VHL disease. However, it was not possible to test for a loss-of-function tumour mutation. It is also feasible that heterogeneity of the tumour means that this result is not truly representative of LOH status.

In the World Health Organisation (WHO) classification of head and neck tumours, adenomatous lesions of the sinonasal cavity are of salivary gland origin [17]. However, benign microcystic lesions similar to the case reported here are not described within the current classification. Due to the rarity of this tumour entity and uncertainty regarding its biological history, categorisation under the current WHO classification is not meaningful. In view of the morphological features, immunoprofile, and similarities to microcystic lesions in the pancreas and previously reported case we feel that the lesion is best described as a microcystic adenoma.

The sinonasal microcystic adenoma described by Xu et al. was surgically resected due to local progression. However, the clinical course of such tumours is unknown [6]. In the case described here, following initial surgical excision, to date three further therapeutic excisions have been required due to local recurrence. The rapid local recurrence is suggestive of a fast-growing lesion, despite the low mitotic index. Therefore, regular imaging surveillance may be indicated, and surgical resection may be appropriate.

## 4. Conclusions

In conclusion, the association of primary microcystic adenoma of the sinonasal cavity with VHL disease is possible but not proven. Such lesions are rare and may provide a considerable diagnostic challenge. Importantly, these lesions are considered essentially benign. However, they may have potential for rapid growth and local recurrence. This suggests that in such cases regular surveillance is merited and that repeated surgical resection may be required.

## Figures and Tables

**Table 1:** Immunohistochemical profile of the renal and sinonasal tumours.

Immunohistochemical marker	Clear cell RCC (2005)	Sinonasal tumour (2012)
CD10	Positive	Negative
RCC	Positive	Negative
CK7	Negative	Positive
CK20	Negative	Positive (patchy staining)
EMA	Positive	Positive
Ki67		Low proliferation
Epithelial marker AE1/3		Positive
Vimentin		Positive
ssms1		Negative
sma		Negative
Alpha-inhibin		Negative
p63		Negative
Thyroglobulin		Negative
NSE		Positive
S100		Negative
GFAP		Negative
PAS		Luminal secretions positive
